# POSA-GO: Fusion of Hierarchical Gene Ontology and Protein Language Models for Protein Function Prediction

**DOI:** 10.3390/ijms26136362

**Published:** 2025-07-01

**Authors:** Yubao Liu, Benrui Wang, Bocheng Yan, Haiyue Jiang, Yinfei Dai

**Affiliations:** 1 College of Computer Science and Technology, Changchun University, Changchun 130012, China; liuyb@ccu.edu.cn (Y.L.); 231501522@mails.ccu.edu.cn (B.W.); 231502539@mails.ccu.edu.cn (B.Y.); 241501500@mails.ccu.edu.cn (H.J.); 2 College of Computer Science and Technology, Jilin University, Changchun 130025, China

**Keywords:** protein function prediction, gene ontology, multiple self-attention mechanisms, protein language model

## Abstract

Protein function prediction plays a crucial role in uncovering the molecular mechanisms underlying life processes in the post-genomic era. However, with the widespread adoption of high-throughput sequencing technologies, the pace of protein function annotation significantly lags behind that of sequence discovery, highlighting the urgent need for more efficient and reliable predictive methods. To address the problem of existing methods ignoring the hierarchical structure of gene ontology terms and making it challenging to dynamically associate protein features with functional contexts, we propose a novel protein function prediction framework, termed Partial Order-Based Self-Attention for Gene Ontology (POSA-GO). This cross-modal collaborative modelling approach fuses GO terms with protein sequences. The model leverages the pre-trained language model ESM-2 to extract deep semantic features from protein sequences. Meanwhile, it transforms the partial order relationships among Gene Ontology (GO) terms into topological embeddings to capture their biological hierarchical dependencies. Furthermore, a multi-head self-attention mechanism is employed to dynamically model the association weights between proteins and GO terms, thereby enabling context-aware functional annotation. Comparative experiments on the CAFA3 and SwissProt datasets demonstrate that POSA-GO outperforms existing state-of-the-art methods in terms of Fmax and AUPR metrics, offering a promising solution for protein functional studies.

## 1. Introduction

Proteins are at the core of life activities and are involved in key processes such as signaling, metabolic regulation, and maintenance of the cellular structure. Defining their functions reveals disease mechanisms and drug targets and is the key to understanding the operation of biomolecules [1]. However, traditional biochemical experiments are costly, lengthy, and low-throughput [2], resulting in reliable functional annotation of only approximately 0.23% of protein sequences [3]. With the rapid development of high-throughput sequencing technology, the number of unannotated proteins has proliferated, and it is difficult to match the experimental validation capability; so there is an urgent need for efficient and accurate protein function annotation methods to break through the experimental limitations [4].

Traditional protein function prediction methods mainly rely on homology-based transfer [5]. For example, BLAST (version 2.13.0) [6] (BLAST: Basic Local Alignment Search Tool) and Diamond [7] (Diamond-Crystal and Molecular Structure Visualization) technologies sequence unknown proteins against known proteins and transfer known protein functions to similar unknown proteins by utilizing the biological principle that homologous proteins tend to have similar functions due to their common evolutionary origins. However, studies have shown that this approach has limited predictive reliability for distant homologous proteins [8].

In recent years, deep learning has shown great potential in protein function prediction, and many computational methods have been developed to solve the protein function prediction problem [9,10]. Graph2GO [11], DeepFMB [12], and Struct2GO [13] integrate multi-source data, such as protein structural domains and amino acid sequences, through graph convolutional networks to infer protein function. At the same time, DeepText2Go [14] utilizes the abstracts of protein-related publications to mine functional information. Despite the progress made by these methods, most of them rely on external experimental data and suffer from high data acquisition costs and poor scalability [15,16]. There are still two significant challenges in deep learning: first, extracting compelling features from amino acid sequences. Transformer-based pre-trained language models (e.g., ProtT5 [17], ESM [18], ProLLaMA [19]) provide efficient solutions. The second is how to map the low-dimensional embeddings of proteins into a large-scale hierarchical functional labelling space. Early approaches used flat classifiers, ignoring semantic associations and hierarchical constraints between labels [20]. In recent studies, DeepGOA [21] captures the topological relationships between labels via a two-layer graph convolutional network (GCN), or TALE [22] learns the low-dimensional representations of labels via matrix decomposition. However, modelling complex semantic relationships between labels still needs to be further optimized to improve the prediction performance [23].

Gene Ontology (GO) [15], as a standard system for protein function annotation, covers three major domains: molecular function (MF), biological process (BP), and cellular component (CC) [24]. GO terms are constructed in the hierarchical directed acyclic graph (DAG), with shallow terms summarizing abstract, generalized semantics and more profound terms focusing on concrete precise semantics [25]. In protein function annotation tasks, each protein is usually associated with multiple GO terms, which makes protein function prediction a large-scale, multi-label classification problem. In addition, the hierarchical structure of GO terms requires consistency in the prediction results. If a protein is annotated as a specific GO term, all the ancestral terms of the term (up to the root node) need to be annotated as well; at the same time, the prediction probability of a specific GO term has to be greater than or equal to the probability of all of its sub-terms [26,27,28]. This hierarchical constraint requires that the model accurately predicts multiple labels and ensures that the prediction results conform to the semantic hierarchy of GO terms.

In order to overcome the limitations of existing methods and solve the problem of multi-label classification for protein function prediction, we conducted an in-depth study. We proposed the end-to-end protein function prediction model, Partial Order-Based Self-Attention for Gene Ontology (POSA-GO). This new protein function prediction method incorporates the GO term topology with the cross-modal attention mechanism. First, protein-level sequence features are extracted using a pre-trained model, which can effectively capture functionally relevant semantic information in individual protein sequences. Second, topological embeddings of GO terms with partial order relations are generated by combining the PO2Vec algorithm [25], explicitly modelling the shortest path dependency between terms to capture the biological information of GO terms better. Finally, the multi-attention mechanism dynamically calculates the association weights of protein features with GO term embeddings to realize context-aware functional annotation and significantly improve prediction accuracy. Comparative experiments on the CAFA3 dataset show that the improved prediction performance is attributed to the combination of high-quality biased-order relation-based feature extraction of GO terms and the joint prediction module of attention, demonstrating the effectiveness of our approach.

## 2. Results

### 2.1. Experimental Setup

We used the same hyperparameter settings to evaluate the POSA-GO model for the three branches of gene ontology: BPO, CCO, and MFO. Among them, GO term embedding is obtained by learning through the GO term encoder: the model is trained for 400 epochs with a batch size of 3000, and the comparative learning objective is optimized using the Adam optimizer (learning rate of 5 × 10^−2^) [29], with the temperature parameter *τ* fixed at 0.1. Negative sampling is set to a total number of samples of k = 80, of which 25% are derived from the ancestor terms of the target term (*u* = 0.25) to balance semantic differentiation with hierarchical relationship maintenance. The POSA-GO model was trained using a uniform base configuration: 25 training cycles, batch size 32, Adam optimizer (learning rate 1 × 10^−4^, weight decay 0.1) with Step-learning rate scheduling (gamma = 0.9) and Dropout (*p* = 0.2) regularization, and the number of multi-attention heads was set to 8. All experiments were executed under randomized seeds of 42, and the hardware platform was NVIDIA RTX 3090 GPUs(NVIDIA Corporation, based in Santa Clara, California, USA.). The model structural parameters were differentially set to address the characteristics of the different GO branches: the BPO branch had a potential dimension of 768 (with an MFO/CCO of 512), and the predicted interim dimensions were 1280 (BPO), 768 (MFO), and 896 (CCO).

Here, POSA-GO is compared with six methods: the label transfer method Naive [30] based on sequence similarity, the homology function prediction Diamond [7] based on homology, the graph convolutional neural network (GCN)-based method DeepGOA [21], the deep learning and sequence comparison method DeepGOPlus [20] based on CNNs, the method based on Transformer TALE [22], and PO2GO [25], a deep-learning method based on improved partial order relations; and in our experiments we use the hyperparameters provided in the original paper.

### 2.2. POSA-GO Outperforms Competing State-of-the-Art Methods

**Figure 1:**
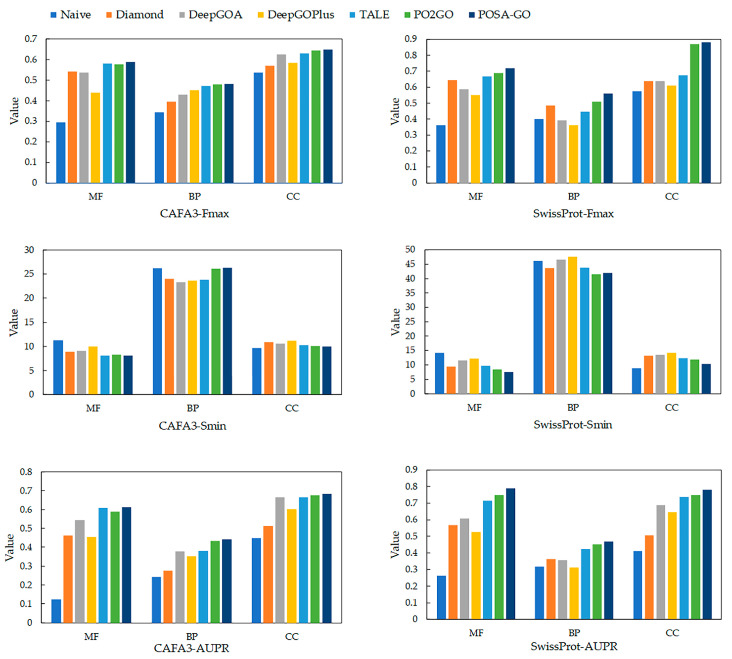
The performance of POSA-GO in predicting protein functions was evaluated against other models using the CAFA3 dataset and the SwissProt dataset across three Gene Ontology (GO) categories: Biological Process (BP), Molecular Function (MF), and Cellular Component (CC). The methods compared include Naive, Diamond, and TALE, which are standalone approaches. DeepGOPlus, PO2GO, and POSA-GO are composite methods. Performance was assessed using three metrics: the area under the precision–recall curve (AUPR), where a higher value is better; minimum semantic distance (Smin), where a lower value is better; and maximum F1-score (Fmax), where a higher value is better.

Figure 1 illustrates the performance of different methods in testing the three GO aspects of the CAFA3 and SwissProt datasets [31]. The GO terms in the biological process (BP) ontology are inherently characterized by a large number of terms, a highly hierarchical structure, a long-tailed distribution of informative terms, and complex cross-branch semantic associations, making the prediction of BP terms significantly more challenging compared with those in the cellular component (CC) and molecular function (MF) ontologies. Traditional and deep-learning methods for BP prediction generally suffer from modeling bias for low-frequency specificity terms and semantic propagation attenuation, leading to difficulties in optimizing information-weighted semantic distance metrics. Thus, Smin usually performs worse in BP prediction than structurally insensitive statistical metrics. Regarding Fmax and AUPR, POSA-GO outperforms other competing methods in all three GOs.

Specifically, in terms of Fmax, POSA-GO outperforms the competing methods in MFO, BPO, and CCO for the CAFA3 and SwissProt datasets by (1.2%, 4.3%), (0.6%, 9.8%), and (0.9%, 1.1%), respectively. AUPR on MFO, BPO, and CCO on the CAFA3 and SwissProt datasets are higher than competing methods by (0.1%, 5.3%), (1.6%, 4.4%), and (1.1%, 4%), respectively. The experimental results of POSA-GO demonstrate that, compared with CNN or GCN-based multi-source fusion methods, features based on the mechanism of multi-head self-attention cross-fertilization exhibit significant advantages.

**Figure 2:**
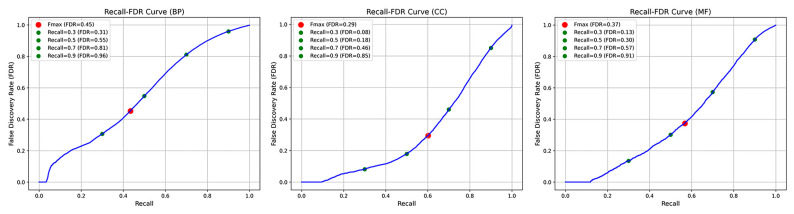
Trends in Recall–FDR for model POSA-GO on the three GO ontologies. As the prediction threshold decreases, the recall increases, accompanied by a significant rise in false discovery rate (FDR), which is evident in the BP and MF ontologies. In the CC ontology, the FDR growth is relatively flat.

In addition, we further analyze the false discovery rate (FDR) of POSA-GO on various ontologies using the CAFA3 dataset and present the Recall–FDR curves in Figure 2 to evaluate the model’s reliability in practical applications. For the BPO model, at maximum recall (approximately 0.9), the false discovery rate reaches as high as 0.959, resulting in a significant number of false positives. In the CCO model, there is improved control of the FDR. At the Fmax threshold of 0.499, the FDR is only 0.295. Even with a high recall of approximately 0.9, the FDR remains below 0.85. In the MFO model, similar to the BPO model, the FDR sharply increases at high recall levels. However, at the optimal threshold of 0.395, the FDR is 0.374, indicating acceptable reliability when predictions are made at the Fmax point. In summary, FDR tends to rise with high recall, but a good balance between recall ability and prediction reliability can be achieved by selecting an appropriate prediction threshold.

### 2.3. The Influence of the Number of Attention Heads on Prediction Performance

We investigated the effect of the number of attention heads (h) on the performance of protein function prediction models in the multi-head self-attention mechanism on the CAFA3 dataset. Four different values of h = 1, 2, 4, and 8 were set for the experiment, and a comprehensive performance evaluation was carried out on three protein function ontologies: MFO, BPO, and CCO. The results are presented in Table 1, which shows that when the value of h ranges from 1 to 4, Fmax exhibits a trend of steady improvement. This indicates that as the number of attention heads increases, the model’s feature extraction ability also steadily improves. It can learn feature representations of different subspaces in parallel, capturing complex information in protein sequences more comprehensively. However, when h increases to 8, the model shows a significant decrease in the Fmax metric on all three tasks and an increase in the Smin metric. This performance degradation can be explained from several perspectives: too many attentional heads can lead to a dimensional explosion of the feature space, increasing the computational overhead of the model, which may produce redundant feature representations and noise interference; the model may also build up too large a sensory field as a result, so that some irrelevant distant dependencies are incorrectly reinforced, interfering with the identification of key local features; too many attentional heads may also lead to the model overfitting, especially in the case of limited training data. Therefore, when applying the multi-head self-attention mechanism, it is necessary to carefully weigh the model’s expressive ability and computational efficiency and set an appropriate h-value.

### 2.4. Model Ablation Study

We conducted ablation experiments on the CAFA3 dataset to evaluate the effectiveness of the GO item embedding module and the joint prediction module based on multi-head self-attention in POSA-GO. Table 2 of the experimental results shows that removing the attention mechanism significantly degrades the model’s performance on all feature categories (MF, BP, CC), as reflected in the overall degradation of the Fmax and AUPR metrics. This phenomenon suggests that the attention mechanism plays a key role in feature interaction and weight assignment to effectively capture the complex association between protein sequences and GO terms. For the GO term embedding module, the performance of the removed model is similarly degraded in terms of the Fmax and AUPR metrics. Note that in the BP task, the AUPR metrics of POSA-GO are slightly lower than those of the versions with attention removed or PO2Vec removed, which may be attributed to the small size of the training data for the BP task under the CAFA3 dataset, which is prone to overfitting when too many parameters are introduced, and the dependence of some of the biological processes on the information of localized regions, which results in the model’s ineffectiveness in capturing the long-distance dependence. In summary, the experimental results verified the advantages of the multi-head self-attention mechanism in feature fusion, which can model long-range dependencies more effectively in cellular components and molecular functions compared with simple feature splicing. Meanwhile, the embeddings obtained through the GO term embedding module training significantly enhance the model’s generalization ability, but the training data size may limit its effect.

## 3. Discussion

Currently, many proteins in existing databases lack functional annotations, and traditional homology-based methods have limited accuracy when predicting the functions of distantly related proteins with low sequence similarity. For proteins with completely unknown functions, prediction methods primarily rely on the intrinsic features of the proteins. Potential functional tags are indirectly inferred by calculating associations between the target protein and annotated proteins that share similar features.

In this study, we propose an innovative protein function prediction model called POSA-GO, which does not require sequence homology comparisons. First, leverage the powerful pre-training capabilities of the protein language model ESM-2 to identify structural features that are evolutionarily conserved and functionally relevant in protein sequences. Next, we employ the PO2Vec algorithm to generate topological embeddings of GO terms with partial order relations, effectively modeling the hierarchical structure and semantic associations between Gene Ontology terms. This enables the model to perform functional inference through the is_a and part_of relationships. Finally, even in the absence of homologous sequences, the cross-modal multi-head self-attention mechanism dynamically establishes the precise associations between protein local features and GO terms, enabling multi-level protein function annotation.

This fusion of sequence features and topological embeddings improves the model’s performance in Fmax and AUPR indexes. It significantly enhances the specificity of functional annotation and the ability to learn with fewer samples. The model’s modular design makes it scalable. It supports the flexible substitution of different protein encoders and its GO term embedding method, which can be extended to other gene ontology learning tasks. It offers a novel technical approach for predicting protein function.

A significant breakthrough in protein structure prediction has been achieved with the introduction of the AlphaFold2 model [32]. This algorithm predicts the 3D structure of proteins based on their amino acid sequences, achieving prediction accuracy at the atomic level [32]. However, this method has limitations. First, its effectiveness is constrained by the availability of 3D structural data. For proteins lacking experimental structural data, prediction errors can impact the accuracy of functional inferences. Second, the relationship between a protein’s structure and its function is closer than that of its sequence; therefore, precise structure-to-function mapping still relies on additional annotation tools. To overcome the limitations mentioned, we can utilize the structural features predicted by AlphaFold2 as complementary inputs, combining them with the sequence features from POSA-GO. A self-attention mechanism is then employed to dynamically establish correlation weights between the feature embeddings and the Gene Ontology (GO) terms. This approach enhances the performance of protein function prediction.

We plan to explore multimodal data fusion and model architectures to optimize POSA-GO in future work. First, inspired by HNetGO [33], introducing a fast and accurate MSA algorithm, diamond, to compute the similarity between protein sequences and protein interaction network data, combined with a dynamic weighting mechanism, may generate a more comprehensive functional characterization. Second, while combining graph neural networks (GNNs) with structural prediction features such as AlphaFold2 [32], literature-derived a priori knowledge maps are introduced to design a cross-graph information transfer strategy based on the attention mechanism, which enables the model to learn both the underlying features from sequence to structure and the high-level functional associations in the literature knowledge. For example, Prot2Text [34] demonstrated that integrating graph and sequence information improves the understanding of protein function. Third, hierarchical-aware contrastive learning strategies can be developed to enhance the semantic differentiation of GO term embedding and design hierarchical attention mechanisms to optimize the interaction process between sequence features and ontology terms, respectively, in addition to focusing on model interpretability enhancement by visualizing the decision basis and constructing memory-enhancing modules to capture rare functional patterns better.

Mass spectrometry (MS)-based proteomics directly identifies and quantifies amino acid sequences, providing an unbiased and systematic analysis of protein expression, modifications, and interactions with high quantitative accuracy, specificity, and cross-species applicability [35]. Notably, MS-based interaction proteomics, such as affinity purification MS (AP-MS), can effectively capture dynamic and condition-specific protein–protein interactions, localize interactions to specific domains, or identify interactions dependent on post-translational modifications (PTMs) [35]. In future work, we propose to use the high-confidence interaction data obtained from AP-MS to improve protein function prediction. We intend to encode the corresponding protein–protein interaction (PPI) network and annotate it with Gene Ontology (GO) terms, which will facilitate transfer learning using graph neural networks (GNNs).

## 4. Materials and Methods

### 4.1. Overview

**Figure 3:**
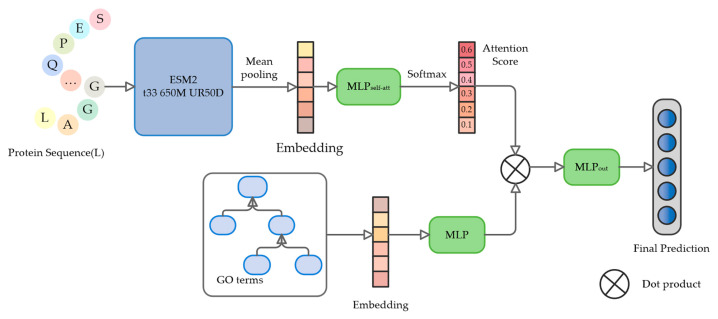
The architecture of POSA-GO. First, the pre-trained language model ESM2 is used to extract the embedded representation of the initial protein, which is then aggregated into the global representation of the protein using a mean pooling strategy. Meanwhile, the GO term embedding module handles the hierarchical topological characterization of gene ontology terms. Then, the global representation of protein sequences and the feature matrices of gene ontology terms are sent in parallel with two MLPs to learn the attention-weight vectors and semantic alignment mappings. Finally, the dot product between protein features and GO term embeddings is dynamically computed through multi-head self-attention mechanisms to derive association weights, and the weighted features are then fed into the MLP output layer to generate the final annotation results.

The POSA-GO model is designed with a modular architecture, which consists of three core components to form a complete prediction process: first, the protein feature extraction module encodes the deep semantic encoding of the input sequences through a pre-trained language model; second, the GO term embedding module specializes in the hierarchical topological features of the gene ontology terms; and lastly, the joint prediction module based on self-attention dynamically integrates the output features from the first two modules that realize end-to-end protein function prediction. The whole system constructs standardized inputs through the input data generation module and outputs the final functional annotation results after being trained by the joint predictor. The framework of the proposed POSA-GO is shown in Figure 3.

### 4.2. Dataset

Gene Ontology (GO) data were obtained from the official GO website, where isolated terms were removed, obsolete term IDs were replaced with their primary IDs, and only the is_a and part_of relationships were retained. Finally, 27,709 terms were obtained in the BPO domain, 11,256 in the MFO domain, and 4043 in the CCO domain. In this paper, we collected, reviewed, and manually annotated proteome sequences from SwissProt [36], which contains 546,651 protein sequences. To demonstrate the generalizability of POSA-GO, this paper uses the same training sequences, experimental annotations, and test benchmarks as the CAFA3 dataset in the literature [25]. It compares it with other protein function prediction methods. The statistics of training and testing sets used in this study are shown in Table 3.

### 4.3. The Architecture of POSA-GO

#### 4.3.1. Pretrained Protein Language Model

In this study, POSA-GO utilizes the protein language model esm2_t33_650M_UR50D (ESM-2 for short, facebookresearch/esm: Evolutionary Scale Modeling (esm): Pretrained language models for proteins) [37] to extract high-quality protein feature representations from amino acid sequences, thereby bypassing computationally intensive multiple sequence alignment (MSA) or structural templates. ESM-2 is a pre-trained protein language model (PLM) based on the Transformer architecture, whose model structure draws on RoBERTa’s [38] improvement strategy and contains a multilayer Self-Attention mechanism and feed-forward neural networks. ESM-2 is designed to provide a high-quality representation of protein features. It is trained through Masked Language Modeling (MLM) combined with Span Masking, which enables it to capture local structure more effectively. During training, the model must predict randomly masked amino acids to learn the contextual dependencies and evolutionary patterns of protein sequences [39]. The training data for ESM-2 come from UniRef50(UniRef | UniProt help | UniProt) [40], which covers over 250 million protein sequences. Compared with the training strategy of ESM-1b [18], ESM-2 employs dynamic mask scaling and gradient accumulation in training to alleviate the overfitting problem in low-complexity regions in protein sequences. The pre-trained ESM-2 can be directly used for downstream tasks without fine-tuning. Specifically, for a protein *p* of length *L* amino acids, the embedding representation of each amino acid is first extracted using ESM-2 and combined into a feature matrix Z∈ℝ^d×^*^L^*, where *d* is the embedding dimension. In order to aggregate the global representation of the protein from the amino acid level embeddings, the same mean pooling strategy as Unsal et al. is used secondly, and the final embedding representation of protein *p* is obtained as *f*(*p*) = *mean*(Z)∈ℝ^d×1^ [41].

#### 4.3.2. Multilayer Perceptron

MLP is a fully connected feed-forward artificial neural network. Its core computation can be expressed as $H^{\left(l\right)}=\sigma \left(H^{\left(l-1\right)}W^{\left(l\right)}+b^{\left(l\right)}\right)$, where $H^{\left(l-1\right)}\in R^{n\times d_{l-1}}$ is the input of the lth layer of the MLP, $H^{\left(l\right)}\in R^{n\times d_{l}}$ is the output of the lth layer, $W^{\left(l\right)}\in R^{d_{l-1}\times d_{l}}$ is the weight matrix of the lth layer, $d_{l}$ denotes the number of neurons in the lth layer, and $b^{\left(l\right)}\in R^{d_{l}}$ is the bias term of the lth layer [42].

#### 4.3.3. Multi-Head Attention Layer

The multi-head attention mechanism in this paper is based on the Transformer architecture, which has been enhanced for protein sequence feature extraction tasks based on previous research [43]. The multi-head attention layer in the model consists of three parts: input projection, parallel attention head computation, and multi-head fusion output. The specific structure is as follows: Unlike the classical Transformer, the query (Q), key (K), and value (V) in POSA-GO use separate input sources to enhance flexibility. The query (Q) is generated from the MLP module (q) by linear transformation and combined with batch normalization, ReLU activation, and Dropout for nonlinear feature extraction to generate a high-latitude semantic representation [44,45,46]. Independent linear layer mapping obtains keys (K) and values (V) to realize the model’s fusion of sequential and non-sequential modal information. Each attention head is aggregated by computing the similarity scores of Q and K and weighting V [43]:

(1) $$\mathrm{Attention}(Q,K,V)=\mathrm{soft}\max\left(\frac{QK^{T}}{\sqrt{d_{k}}}\right)V,$$

where the scaling factor $\sqrt{d_{k}}$ is used to prevent the gradient from being destabilized by too large a dot product result. The model uses multiple parallel attention heads, and the outputs of each head are spliced and fused through a non-dynamic quantized linear layer, omitting the feed-forward network module in classical multi-head attention and plugging the attention outputs directly into the downstream prediction task [43]. Multi-head attention is defined as [43]

(2) $$\mathrm{MultiHead}(Q,K,V)=\mathrm{Concat}({\mathrm{head}}_{1},\ldots {\mathrm{head}}_{n})W^{O},$$

where n is the number of attention heads, and $W^{O}\in R^{d_{k}\times d}$ denotes the output projection matrix for fusing the outputs of all heads. ${\mathrm{head}}_{j}$ is defined as [43]

(3) $${\mathrm{head}}_{j}=\mathrm{Attention}\left(Q{W_{j}}^{Q},K{W_{j}}^{K},V{W_{j}}^{V}\right),$$

where ${W_{j}}^{Q}\in R^{d_{k}\times d}$, ${W_{j}}^{K}\in R^{d_{k}\times d}$, and ${W_{j}}^{V}\in R^{d_{k}\times d}$ are the jth header query, key projection, and value projection matrices, respectively, where d is the bit size of the hidden embedding vector and $d_{k}=\frac{d}{n}$ [43].

#### 4.3.4. GO Term Embedding with PO2Vec

Protein function prediction is a multi-label classification problem. In most approaches, the classes (GO terms) are constructed as a directed acyclic graph (DAG), and this structure has consistency requirements for the final prediction results [47,48,49]. Here, the model applies PO2Vec proposed by [25] to thoroughly learn the structural information between GO terms using a biased ordering relation within/outside the paths. This learning strategy defines two constraints—(1) in-path: for a given term $t_{b}$, its ancestor term $t_{p}$, and another in-path term $t_{f}$, if $t_{p}$ is closer to $t_{b}$ than $t_{f}$, then $\mathrm{len}(\mathrm{sp}(t_{b},t_{p}))\leq \mathrm{len}(\mathrm{sp}(t_{b},t_{f}))$, where $\mathrm{len}(\mathrm{sp}(t_{b},t_{p}))$ returns the number of edges of the shortest path between the terms, i.e., the similarity between $t_{p}$ and $t_{b}$ is more significant than that between $t_{b}$ and $t_{f}$. (2) out-path: For a given term $t_{b}$, its ancestor term $t_{p}$, and out-of-path term $t_{f}$, there exists a similarity between $t_{b}$ and $t_{p}$ that is much greater than between $t_{b}$ and $t_{f}$. Note that given two terms $t_{b}$ and $t_{p}$, if there is a direct or indirect ancestor or descendant relationship between $t_{b}$ and $t_{p}$, then $t_{b}$ is an in/out-of-path term for $t_{p}$. Otherwise, $t_{b}$ is an out-of-path term for $t_{p}$, i.e., $\mathrm{len}\left(\mathrm{sp}\left(t_{b},t_{p}\right)\right)=+\infty$.

For GO term feature embedding, PO2Vec employs an unsupervised representation learning method, contrast learning, to maximize the consistency between similar instances and minimize the consistency between different instances. The commonly used loss function InfoNCE is as follows [50]:

(4) $$L_{N}=-\underset{X}{Ε}\left[\log\frac{f\left(x,x^{+}\right)}{\sum_{x_{p}\in X}f\left(x,x_{p}\right)}\right],$$

where X is the within-batch training sample, $x^{+}$ is a positive sample, and $f\left(x,x_{p}\right)$ is the similarity between x and $x_{p}$.

Traditional stochastic negative sampling in modelling results in models biased toward learning simple, irrelevant terms and ignoring important but difficult term associations because there are far more out-of-path terms than in-path terms. For this reason, an improved harmful sampling method is employed to increase the proportion of in-path terms, prompting the model to learn to distinguish between semantically similar terms and thus learn hierarchical, biased order relations more accurately. The method is divided into three stages: index construction, hierarchical sampling, and comparison learning.

First, three types of candidate sets for each term $t_{i}$ are constructed: in-path term list ${Ζ}_{in}(t_{i})$, out-of-path term list $Ζos(t_{i})$, and cross-domain out-of-path term list ${Ζ}_{od}(t_{i})$. ${Ζ}_{in}(t_{i})$ contains terms that are on the same path as $t_{i}$ (e.g., ancestor or descendant nodes), $Ζos(t_{i})$ contains terms that belong to the same domain as $t_{i}$ but are not on its path, and ${Ζ}_{od}(t_{i})$ contains terms that belong to different domains and are not path-associated with $t_{i}$, where the terms in ${Ζ}_{in}(t_{i})$ are sorted in ascending order of the shortest path length.

Second, high-quality negative samples are proportionally drawn from the three types of lists to balance semantic similarity and difference. Specifically, an ancestor term from ${Ζ}_{in}(t_{i})$ is randomly selected as a positive sample ${t_{i}}^{+}$, and the total number of negative samples is fixed as k. The proportion of negative samples within a path is controlled by the hyperparameter u. The in-path negative samples $N_{in}\left(t_{i}\right)$ are obtained by sampling $min(k\times u,|{Ζ}_{in}(t_{i})|)$ terms from ${Ζ}_{in}(t_{i})$, whose path lengths are all larger than $len\left(sp\left(t_{i},{t_{i}}^{+}\right)\right)$, to help the model distinguish fine-grained hierarchical relationships. The out-of-path negative samples $N_{out}\left(t_{i}\right)$ are sampled from $Ζos(t_{i})$ and ${Ζ}_{od}(t_{i})$ by $k-|N_{in}\left(t_{i}\right)|$ to enhance the model’s judgment of cross-domain and irrelevant terms.

Finally, the sampled positive and negative samples are used to train the contrast loss, pulling $t_{i}$ closer to the representation of the positive samples ${t_{i}}^{+}$ and pushing $t_{i}$ farther away from the representation of the negative sample set $N_{in}\left(t_{i}\right)\cup N_{out}\left(t_{i}\right)$. We apply the balanced InfoNCE loss function to learn the embedding representation of GO terms:

(5) $$L_{GO}=-\sum_{i=1}^{m}\log\frac{f(e(t_{i}),e({t_{i}}^{+}))}{f(e(t_{i}),e({t_{i}}^{+}))+B_{GO}},$$

(6) $$B_{GO}=\frac{k}{2}\left(\frac{1}{\left|N_{in}(t_{i})\right|}\sum_{t_{j}\in N_{in}(t_{i})}f(e(t_{i}),e(t_{j}))+\frac{1}{\left|N_{out}(t_{i})\right|}\sum_{t_{j}\in N_{out}(t_{i})}f(e(t_{i}),e(t_{j}))\right),$$

(7) $$f(e(t_{i}),e(t_{j}))=\frac{\exp(e(t_{i}{)}^{Τ}\cdot e(t_{j}))}{\tau },$$

where τ is a temperature hyperparameter to control the f(⋅,⋅) function, τ is smaller so that similarity differences are amplified, τ is larger so that the sample distribution is smoother, e(⋅) denotes the embedding vector of the samples, and $B_{GO}$ balances the effect of the number of negative samples in different categories.

#### 4.3.5. Protein-Term Link Prediction

For any given protein sequence and GO term, we generate initial embeddings of the sequence and GO term, respectively, by pre-training, project both into the same semantic space using two completely independent MLPs to eliminate distributional differences, subsequently compute the dot product of the projection vectors of the protein with all the terms as the base similarity, and output the final prediction by a third MLP with a nonlinear transformation of the similarity vectors probability (Figure 1).

Specifically, for a given protein $p_{i}$, its original embedding representation $f\left(p_{i}\right)$ is obtained through the protein feature extractor ESM-2. For a given Gene Ontology (GO) term $t_{j}$, its original embedding $e\left(t_{j}\right)$ is obtained through the terms encoder. In order to map the embeddings of proteins and Gene Ontology (GO) terms into the same semantic space, two independent multilayer perceptron machines (MLPs) are used to perform the nonlinear transformation, respectively:

(8) $$f_{proj}\left(p_{i}\right)=MLP_{protein}\left(f(p_{i})\right),$$

(9) $$e_{proj}(t_{j})=MLP_{GO}(e(t_{j})),$$

where the original protein embedding $f(p_{i})\in R^{d}$ has dimension d, the projected protein embedding $f_{proj}\left(p_{i}\right)\in R^{k}$ has dimension k, and the projected Gene Ontology embedding $e_{proj}(t_{j})\in R^{k}$ is consistent with the protein embedding dimension. We do this by computing the similarity vector $s\in R^{m\times 1}$ of protein $p_{i}$ with all GO terms ${{\left\{t_{j}\right\}}^{m}}_{j=1}$:

(10) $$s_{j}=e_{proj}(t_{j}{)}^{T}\cdot f_{proj}\left(p_{i}\right),$$

which reacts to the similarity between the two, where m is the number of Gene Ontology terms. Unlike traditional methods that directly use the similarity s as the prediction result, we invoke another MLP to optimize the similarity vector further:

(11) $$\hat{y}=MLP_{pred}(s),$$

where the predicted probability vector $\hat{y}\in {\left[0,1\right]}^{m}$, and each element ${\hat{y}}_{j}$ represents the probability that the protein is annotated as $t_{j}$. Since a protein may correspond to multiple Gene Ontology terms, a binary cross-entropy loss is used here:

(12) $$L_{PRED}=-\sum_{j=1}^{m}\left(y_{j}\;log\;{\hat{y}}_{j}+(1-y_{j})\log(1-{\hat{y}}_{j})\right),$$

where $y_{j}\in \left\{0,1\right\}$ indicates whether the protein is authentically annotated with the Gene Ontology term $t_{j}$.

### 4.4. Evaluation Metrics

Three metrics are used in this study to evaluate the predictive performance of the model, namely Fmax, Smin, and AUPR. Fmax is the maximum of all F1 scores computed at different prediction thresholds $\left\{0,0.01,0.02,\ldots ,0.99,1\right\}$, as defined below [51,52]:

(13) $$F_{max}=\underset{\tau }{max}\left\{\frac{2\times \mathrm{Pr}\left(\tau \right)\times Rc\left(\tau \right)}{\mathrm{Pr}\left(\tau \right)+Rc\left(\tau \right)}\right\},$$

where $\mathrm{Pr}\left(\tau \right)$ and $Rc\left(\tau \right)$ are the precision and recall of the prediction results for all proteins under the threshold τ, respectively defined as follows:

(14) $$\mathrm{Pr}\left(\tau \right)=\frac{1}{q\left(\tau \right)}\sum_{i=1}^{q\left(\tau \right)}\frac{\left|P_{i}\left(\tau \right)\cap T_{i}\right|}{\left|P_{i}(\tau )\right|},$$

where $q\left(\tau \right)$ is the number of proteins with annotations whose prediction score is greater than or equal to τ. $T_{i}$ and $P_{i}\left(\tau \right)$ are the set of accurate annotations for protein i and predicted annotations for the ith protein at threshold τ, respectively. |⋅| denotes the number of elements. q is the total number of proteins used for evaluation.

(15) $$Rc(\tau )=\frac{1}{q}\sum_{i=1}^{q}\frac{\left|P_{i}(\tau )\cap T_{i}\right|}{\left|T_{i}\right|},$$

Smin is used to measure the minimum semantic distance between the predicted annotations and the accurate annotations, reacting to the degree of functional semantic proximity between the predicted results and the accurate annotations, as defined below [24]:

(16) $$S_{min}=\underset{\tau }{min}\left\{\sqrt{ru(\tau {)}^{2}+mi(\tau {)}^{2}}\right\},$$

(17) $$ru(\tau )=\frac{1}{q}\sum_{i=1}^{q}\sum_{c\in T_{i}-P_{i}\left(\tau \right)}ic(t),$$

(18) $$mi(\tau )=\frac{1}{q}\sum_{i=1}^{q}\sum_{c\in P_{i}\left(\tau \right)-T_{i}}ic(t),$$

(19) $$ic(t)=-\log\left(\mathrm{Prob}(t|\mathrm{Pa}(t))\right),$$

where $ic\left(t\right)$ is the information content of Gene Ontology term t, $ru\left(\tau \right)$ measures the average information content of terms that are true but not predicted, $mi\left(\tau \right)$ measures the average information content of terms that are incorrectly predicted, Prob(t|Pa(t)) is the conditional probability of Gene Ontology term t appearing under the condition of a given parent term, which is used to reflect the rarity of the term, and Pa(t) is the set of parent terms of Gene Ontology term t [24]. AUPR is the area enclosed by the Precision–Recall curve with the area of the region enclosed by the coordinate axes, which is more comprehensive than the metrics under a single threshold and reflects the overall performance of the model under all thresholds.

## 5. Conclusions

In summary, the model POSA-GO proposed in this study significantly improves the accuracy of functional annotation on the benchmark test set. The core innovations of the model are reflected in the following: firstly, the cutting-edge protein language model ESM-2 is used to deeply mine the functional information in sequences; secondly, the discrete GO terms are modelled as continuous topological embeddings based on the bias–order relationship [52]; and lastly, the precise alignment of sequence features and functional semantics is achieved by the multimodal fusion mechanism. This study provides a reliable research solution for the functional annotation of proteins.

## Figures and Tables

**Table 1:** The performance of POSA-GO with different h on the CAFA3 dataset.

Number of Attention Heads	Fmax			Smin			AUPR		
	MF	BP	CC	MF	BP	CC	MF	BP	CC
h = 1	0.582	0.48	**0.645**	**8.138**	**25.695**	**10.063**	0.596	0.444	0.673
h = 2	**0.583**	**0.483**	**0.645**	8.172	25.812	10.099	**0.598**	0.443	**0.675**
h = 4	0.581	0.482	**0.645**	8.254	25.858	10.096	0.594	0.442	0.672
h = 8	0.580	0.481	0.644	8.26	25.818	10.08	0.595	**0.445**	0.673

Note: Best performance in bold Fmax and AUPR, highest; Smin, lowest.

**Table 2:** Ablation analysis: Effect evaluation on the CAFA3 dataset.

Method	Fmax			Smin			AUPR		
	MF	BP	CC	MF	BP	CC	MF	BP	CC
POSA-GO w/o attention	0.577	0.478	0.644	8.324	**26.135**	10.116	0.592	0.435	0.675
POSA-GO w/o PO2Vec	0.571	0.469	0.641	8.381	26.142	10.155	0.59	0.432	0.678
POSA-GO	**0.589**	**0.481**	**0.65**	**8.129**	26.312	**10.029**	**0.611**	**0.442**	**0.683**

Note: Best performance in bold Fmax and AUPR, highest; Smin, lowest.

**Table 3:** The number of protein sequences with experimental annotations in CAFA3 and SwissProt datasets grouped by sub-ontologies.

	Statistics	BP	MF	CC
CAFA3	Training Set	50,813	35,086	49,328
	Testing Set	2133	1088	1094
	Number of annotations	19,901	6367	2470
SwissProt	Training Set	49,003	36,403	47,177
	Testing Set	5402	4038	5165
	Number of annotations	19,832	6785	2760

## Data Availability

The datasets analyzed in this study are from the SwissProt dataset: ‘http://www.uniprot.org/uniprot/’ accessed on 10 May 2023. and the CAFA3 dataset: ‘https://github.com/xbiome/protein-annotation’ accessed on 16 May 2024.
